# To regenerate or not to regenerate: Vertebrate model organisms of regeneration‐competency and ‐incompetency

**DOI:** 10.1111/wrr.13000

**Published:** 2022-02-22

**Authors:** Can Aztekin, Mekayla A. Storer

**Affiliations:** ^1^ School of Life Sciences Swiss Federal Institute of Technology Lausanne (EPFL) Lausanne; ^2^ Department of Physiology, Development and Neuroscience and Wellcome‐MRC Cambridge Stem Cell Institute University of Cambridge Cambridge

## Abstract

Why only certain species can regenerate their appendages (e.g. tails and limbs) remains one of the biggest mysteries of nature. Unlike anuran tadpoles and salamanders, humans and other mammals cannot regenerate their limbs, but can only regrow lost digit tips under specific circumstances. Numerous hypotheses have been postulated to explain regeneration‐incompetency in mammals. By studying model organisms that show varying regenerative abilities, we now have more opportunities to uncover what contributes to regeneration‐incompetency and functionally test which perturbations restore appendage regrowth. Particularly, *Xenopus laevis* tail and limb, and mouse digit tip model systems exhibit naturally occurring variations in regenerative capacities. Here, we discuss major hypotheses that are suggested to contribute to regeneration‐incompetency, and how species with varying regenerative abilities reflect on these hypotheses.

**Abbreviations**
AEC: apical‐epithelial‐cap
AER: apical‐ectodermal‐ridge
Mc4r: melanocortin 4 receptor
NF: Nieuwkoop and Faber
scRNA‐Seq: single‐cell mRNA sequencing
SDF1‐a: cytokine stromal derived factor 1a

## 1 INTRODUCTION

Appendage regeneration has fascinated biologists for centuries,^1^ as uncovering the molecular and cellular players regulating this process holds potential for therapies aimed at regenerating human limbs. While there are many different model organisms (e.g. zebrafish, salamander, deer) and appendage regeneration models (e.g. tail, digit tip, antler), each with unique characteristics, the bulk of our understanding of appendage regeneration has been derived from studies investigating amphibian limb and tail regeneration.^2^ Amputations of appendages in these animals induce certain lineages to show a morphologically identified dedifferentiation phenotype and co‐currently a simple wound epidermis covers the amputation plane.^3^, ^4^ This simple wound epithelium progresses into becoming a specialised wound epithelium called the apical‐epithelial‐cap (AEC). The AEC then functions as the key signalling centre during regeneration enabling expansion of lineage‐restricted stem and progenitor populations, that are collectively termed the blastema.^5^, ^6^, ^7^, ^8^ The continuous interaction between the AEC and the blastema restores the lost appendage. Meanwhile, amputation of the mammalian limb fails to form an AEC or a blastema, and instead results in fibrotic scar formation and regenerative failure.^9^

Although mice, and in general amniotes including chickens^10^ and lizards,^11^ cannot perform limb regeneration, mouse digit tip amputations result in regrowth through formation of a blastema.^12^, ^13^ Interestingly, unlike limb regeneration in amphibians, there is no reported evidence that an AEC forms during digit tip regeneration, although the wound epidermis is suggested to be important for digit tip regrowth.^7^ Instead of a signalling center AEC, nail bed stem cells were proposed to act as a signalling center population enabling expansion of progenitor cells during digit tip regeneration.^14^, ^15^ Nonetheless, it remains unclear why mammals, and amniotes in general, cannot perform full limb regeneration.

Correlative studies using regenerating (e.g. axolotl, zebrafish) species have led to the inference of multiple hypothesis to explain why regenerative capacity is limited in mammals. These include the quantity of nerve connections,^16^, ^17^ metabolic and nutritional demands,^18^ regulatory element repurposing,^19^ bioelectrical changes,^20^ immune system regulation^21^ and intrinsic and extrinsic factors required for cellular plasticity.^22^, ^23^ Nonetheless, while we have learnt a lot about how regeneration can occur from model organisms without any impediments to regeneration, these animals cannot help us test strategies to restore regenerative potential. Species that exhibit naturally occurring regeneration‐competent and ‐incompetent conditions enable the discovery of natural causes of the loss of appendage regeneration ability, and testing the sufficiency of perturbations to induce regeneration. For this, *Xenopus laevis* limb and tail, and mouse digit tip regeneration models serve as excellent systems for addressing the translational components of regenerative studies. Both organisms exhibit a robust immune response to injury and regeneration of these appendages requires multiple tissue types including skin, nerves, blood vessels and bone/cartilage to regrow in a temporally and spatially precise manner to replace the original structure. Additionally, mouse digit tip regeneration represents a clinically relevant mammalian model of regeneration, mimicking the response observed in human fingertip injuries.^13^, ^24^, ^25^, ^26^

Here, we will focus on the impact of immune system regulation, and the inability to generate or mobilise cells and form structures required for regeneration as underlying reasons for vertebrate appendage regeneration‐incompetency, as much of the literature targets these topics. While doing this, we will ask how model organisms, particularly *Xenopus* limb and tail, and mouse digit tip systems, with varying regeneration levels reflect on these hypotheses, and share our perspectives on the potential of mammalian limb regeneration.

### 1.1 *Xenopus laevis* tail and limb, and mouse digit tip as model systems to study regeneration‐incompetency

Before proceeding further, it is critical to briefly detail how *Xenopus laevis* and mice regrow their lost appendages.

**FIGURE 1:**
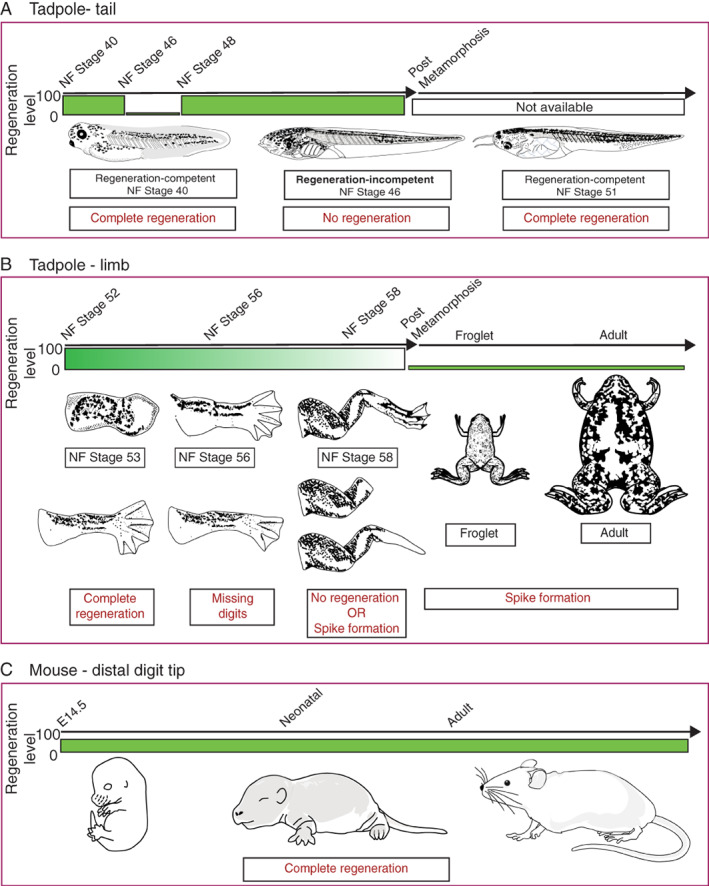
Selected model organisms of regeneration‐competency and regenerationincompetency. (A) (Top) Xenopus laevis tadpoles can regrow their tails throughout their life before metamorphosis, except for a brief period (NF Stage 46 and 47) where amputations result in no regeneration. (Bottom) Example schematics depicting the tadpole morphology and their regeneration status. (B) Xenopus laevis tadpoles can regrow their limbs but lose this ability during their development. (Bottom—Left) Example schematics depicting the tadpole limb morphology and amputation outcomes. (Bottom—Right) Schematics describing differences in froglets and adults and their amputation outcome. (C) (Top) Mice can regrow their lost distal digit tips throughout their life. (Bottom) Schematics for animal morphologies. Green bars indicate the level of regenerative capacity with a wide green bar demonstrating that the animal is regeneration competent. Small bars indicate a loss of regenerative capacity and the fading green bar represents the gradual reduction in regenerative ability

*Xenopus laevis* tadpoles can regrow lost tails and its components (e.g. spinal cord, notochord) during development but lose this ability transiently at certain developmental stages (Nieuwkoop and Faber [NF], 1956; Stage 46–47), termed the ‘refractory period’^27^ (Figure 1A). During the refractory period, tadpoles do not form an AEC.^28^ Instead, a simple wound epithelium covers the amputation plane during these developmental stages, but no subsequent growth is observed. Interestingly, amputating tails during the refractory period results in a non‐regenerative stump tissue, however amputating this stump tissue in the post‐refractory period results in regeneration.^27^ These results may indicate a potential problem with the initial regeneration‐promoting signals. Moreover, amputating tails more anteriorly or posteriorly does not impact the regenerative outcome, although they may exhibit different tissue growth kinetics.^29^ The ability to regenerate tails seems to be conserved phenomenon among different frog species (e.g. *Xenopus tropicalis*,^30^ *Rana pipiens* ^31^). Nonetheless, the refractory period was characterised approximately 20 years ago only in *Xenopus laevis*, and its presence/absence in other species such as *Xenopus tropicalis* has only recently started to be discussed.^32^, ^33^

**FIGURE 2:**
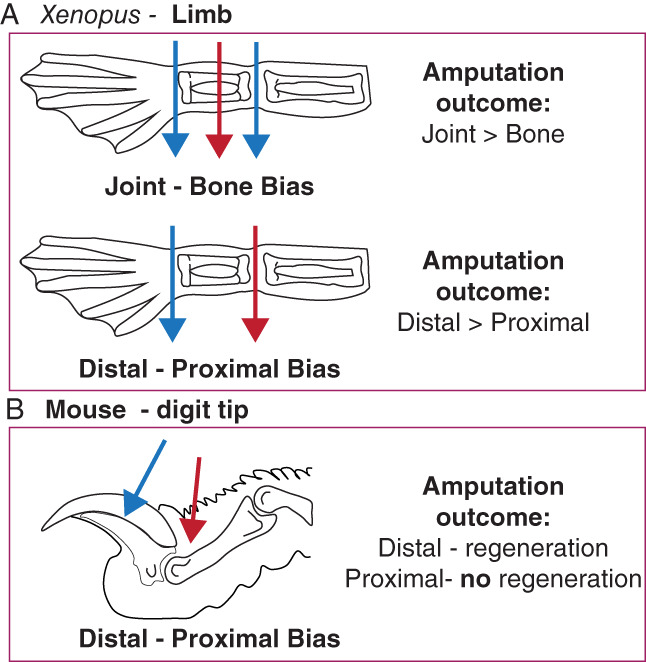
Xenopus limb and mouse digit tip regeneration has positional biases. (A) (Top) Xenopus limb amputations (NF Stage 54–58) to bone result in greater deficits in regeneration (e.g. more missing digits) compared to joint amputations. (Bottom) Xenopus limb amputations (NF Stage 54–58) to proximal regions, which remove more of the limb, result in worse regenerative outcomes (e.g. more missing digits) compared to distal amputations. (B) Mice can regrow distal digit tips, but fail to regenerate upon amputations that remove the nail entirely. Throughout the figure, red arrows indicate the amputation positions resulting in less or no regeneration, compared to blue arrow indicated positions

In contrast to tail regeneration, *Xenopus laevis* progressively lose their ability to regenerate limbs during their development^34^ (Figure 1B). Amputating developing *Xenopus* tadpole limb buds at early stages of growth (NF Stage 52–54) results in perfect regeneration of the limb including all 5 digits. This ability has been attributed to being a developmental continuity. Still, the same phenotype is not seen in chicken and lizard limb bud amputations,^11^, ^35^, ^36^ highlighting that *Xenopus* limb buds display regenerative abilities. When tadpole limbs are in their secondary phase of growth (NF Stage 56–57), amputations result in restricted regeneration, whereby only 2–3 digits are regenerated. Towards metamorphosis (NF Stage 58–60), amputations result in simple wound healing or spike formation. Interestingly, tadpole limb regeneration also shows differences depending on the amputation position^22^, ^34^, ^37^, ^38^ (Figure 2A). Amputations to joint regions result in better regenerative‐outcomes, compared to amputations to bones. Moreover, proximal amputations result in worse regenerative outcomes compared to distal amputations. Unlike the late‐stage pre‐metamorphic tadpole hindlimb regeneration model, regenerative‐growth is assessed in forelimbs in post‐metamorphic froglets, and they uniformly regrow a spike. The growing spike is mainly an unpatterned cartilaginous rod that lacks muscles and joints.^39^, ^40^ Furthermore, the same spike formation phenotype is maintained when froglets reach sexual maturity and in adult frogs.^3^, ^34^ Based on current studies, similar progressive loss of regeneration‐competency is observed in wide‐range of anurans (e.g. *Xenopus tropicalis*,^41^ *Lepidobatrachus laevis* ^42^).

Unlike *Xenopus laevis*, mice cannot regrow their limbs or tails, but they can regrow the ends of their digit tips. In this model organism regenerative capacity is linked with the plane of amputation and is not influenced by the developmental age of the animal. Removal of the digit tip distal to the nail bed in embryonic,^43^ peri‐natal^44^ and adult mice^12^, ^24^, ^45^ elicits a multi‐step regenerative process **(**Figure 1C **)**. This process first involves a wound healing response and epidermal closure, followed by the formation of the blastema, a heterogenous population of cells that forms between the injured bone and newly‐formed epidermis. The cells of the blastema then co‐ordinately regenerate the vasculature, bone, dermis and loose connective tissue. Alternatively, amputations that occur more proximally, or that remove the nail bed, fail to regenerate^13^, ^24^, ^46^ **(**Figure 2B **)**. Here, the injury response initiates a series of events that is analogous to bone fracture repair including formation of cartilaginous callus that caps the severed bone and subsequent bone remodelling.^47^ This level dependent regenerative response indicates that the nail is an important structure necessary for mounting a regenerative response. This observation is further supported by experiments that transplanted the nail onto proximal amputations, inducing ectopic bone growth,^48^ together with an elegant study demonstrating that canonical Wnt signalling in the nail epidermis plays an important role in directing the response of mesenchymal cells during digit tip regeneration.^15^ However, while these studies highlight the importance of the nail, neither nail transplantation or ectopic induction of Wnt signalling in the epidermis was sufficient to fully overcome and explain regeneration‐incompetency in proximal amputations.

### 1.2 Immune system responses are one of the key contributors to regeneration‐incompetency

The immune system plays an important role in orchestrating tissue repair and regeneration. Indeed, the type of immune response, its duration and the type of cells involved are crucial in determining the outcome of the healing process. In response to damage, tissue regeneration initiates a multi‐phasic immune response. The first phase involves components of the innate immune system which comprises neutrophils, monocytes and macrophages with their polarised M1 inflammatory and M2 anti‐inflammatory states.^49^ These cells have been shown to play a number of different roles including promoting haemostasis, re‐organising the extra‐cellular matrix, clearing senescent cells, and promoting other environmental changes that enable the AEC and blastema to form.^50^, ^51^, ^52^ The innate immune response is then followed by the activation of the adaptive immune system. While less is known about how cells of the adaptive immune system influence tissue regeneration, it has recently been shown that regulatory T cells limit inflammation‐induced tissue damage in zebrafish and can promote stem cell proliferation and differentiation during lung repair.^53^, ^54^

Since we know that the immune response is important for tissue regeneration, could differences in immune system complexity, composition and regulation account for regeneration‐incompetency in mature *Xenopus laevis* and mice? Several major hypotheses have emerged.

#### 1.2.1 Immune system complexity is detrimental to regenerative capacity

Differences in immune system complexity across species have long been associated with the loss of regeneration competency. In particular, highly regenerative species have been observed to have a more primitive adaptive immune system compared to regeneration‐incompetence animals. For example, mature frogs and mice have a complex immune system with both innate and a diverse range of adaptive immune responses that are initiated swiftly in response to injury.^55^, ^56^ By comparison, salamanders have a strong innate immune system however lack several adaptive immune responses including a failure to elicit a detectable reaction to soluble antigens^57^ and lack acute xenograft rejection responses.^58^ A recent study, however, has investigated whether regeneration‐incompetency in mammals is directly related to the presence of a strong adaptive immunity and inflammatory response. By characterising the immune response during regeneration in spiny mice compared to fibrotic healing in *mus musculus*, it was demonstrated that regenerative processes produced a stronger and more prolonged adaptive immune response.^59^ This suggests that an adaptive immune response is not antagonistic to regeneration.

#### 1.2.2 Maturation of the immune system suppresses regenerative capacity

The second hypothesis to explain the loss of regenerative ability across species is that as the immune system matures, the capacity to regenerate appendages declines. This idea is well supported by observations that the progressive loss of *Xenopus* limb regeneration coincides with developmental age and subsequent maturation of its immune system. In *Xenopus* laevis tadpoles, local treatment with anti‐inflammatory small molecules improves limb amputation outcomes in older tadpoles that are regeneration‐restricted.^60^, ^61^ It is important to note here that these perturbations do not seem to revert regeneration‐incompetency to ‐competency completely, indicating that suppression of the inflammatory response can only partially rescue regenerative capacity. Conversely, increasing inflammation by treating regenerating limbs with local BeSO4 reduces limb regeneration ability and hinders blastema formation.^60^, ^62^

In mammals it is widely believed that loss of regenerative ability evolved with the need for a strong adaptive immune system to combat harmful pathogens. This idea is supported by the observation that in rodents the transition from scarless wound healing/skin regeneration early in gestation to a scarring response late in gestation is accompanied by an increase in the level of inflammation as the immune system develops.^52^, ^63^ However, this phenomenon, nor immune system complexity, cannot account for endogenous regeneration models in adult mammals because how is it possible to forfeit regenerative ability systemically yet retain it in select regions of the body such as the distal digit tip? Instead, it is plausible that it is the tight regulation of a multiphasic immune response that is indispensable for successful regeneration of tissues.

#### 1.2.3 Regulation of the immune system in the local tissue microenvironment

Unlike *Xenopus* limb regeneration, *Xenopus* tail and several mammalian models including mouse digit tip regeneration highlights that the local regulation of the immune system is the key for regenerative success.

In the model system of *Xenopus laevis* tadpole tail regeneration, where regenerative abilities are lost for a transient period, it has been shown that anti‐inflammatory drugs can induce tail regeneration during the refractory period.^64^ Moreover, signalling pathways closely associated with macrophage polarisation, such as hydrogen pump activity,^65^ reactive‐oxygen‐species,^66^, ^67^ oxygen influx^66^ or changes in bacterial content^68^ can also restore tail regeneration ability. Additionally, perturbations to these pathways in regeneration‐competent tadpoles block tail regrowth. The above‐listed tail perturbations can induce regeneration in most tadpoles; nonetheless, in these experiments, there have been animals that fail to exhibit restoration of regeneration. It remains unclear why tadpole‐specific differences are seen and whether these are due to technical problems or biological differences between individual animals.

Recent single‐cell mRNA sequencing (scRNA‐Seq) based assessment further highlighted that tail regeneration‐competent and regeneration‐incompetent tadpoles harbour a very similar set of cell types.^50^ However, how these cells respond to amputation is significantly different, particularly the response of the innate immune cells. Upon amputation, the regeneration‐competent tadpoles that can form an AEC contains more M2‐like reparative and anti‐inflammatory myeloid cells whereas regeneration‐incompetent tadpoles that cannot form an AEC recruit more M1‐like inflammatory myeloid cells at the amputation plane. Moreover, activation of these anti‐inflammatory myeloid lineage cells is associated with environmental changes resulting in successful AEC formation during tail regeneration. Although the underlying reasons for differential immune system activation remains unclear, the tail regeneration model could be an ideal system to investigate molecules that boost M2‐like reparative and anti‐inflammatory programmes.

Extensive studies in mammals describe the effects of immune cells in non‐regenerating wounds.^69^, ^70^ Nonetheless, our knowledge of the immune response during mammalian regeneration is poor. Recent studies have demonstrated, however, that much like regeneration in salamanders, frogs and fish, macrophages play an essential role in promoting successful regeneration in the neonatal mouse heart,^71^ adult spiny mouse ear pinna,^72^ adult mouse digit tip.^73^ For example, when macrophages were depleted following distal digit tip amputations, subsequent wound closure, blastema formation and regeneration was inhibited.^73^ These results indicate that macrophages play an essential role in the transition from wound healing to regeneration during vertebrate regeneration however how this response is regulated is not yet understood. In the digit tip, it could be speculated that the nail organ, which is necessary for a regenerative response, modulates acute inflammation produced by amputation injuries. Indeed, examination of human nail matrix histologically has shown that this tissue expresses a wide variety of anti‐inflammatory and immunosuppressive proteins^74^ and matrix derived from amputated patient samples has been further shown to stimulate macrophage polarisation towards a pro‐healing phenotype *in vitro*.^75^ While these observations are promising, studies investigating immune cell interactions with the nail organ in vivo will provide important insights as to the validity of these speculations.

Despite these correlative observations and functional perturbations that influence regeneration, several critical points remain unclear. First, upon injury do amphibians recruit completely different immune cells than amniotes? If not, how are these immune cells regulated in a way that promotes regeneration rather than inhibiting it? Indeed, in contrast to mammals, where immune cells derived from the blood come predominately from the bone marrow, the pro‐regenerative cells of the immune system in the axolotl derive from the liver and spleen to contribute to regeneration.^76^ This demonstrates that differences in immune cell regulation are present between regeneration competent and ‐incompetent species and may in part account for differing regenerative abilities. Additionally, scRNA‐seq based studies from other species can serve as valuable resources to compare similarities among myeloid lineage cells across species. Beyond transcriptional comparisons, recent work identified species‐specific post‐transcriptional differences between mouse and axolotl macrophages.^77^ Whether these changes account for functional differences in regenerative capacity requires further investigation. Aquatic animals may have certain advantages over terrestrial animals. Notably, it might be possible that the environmental pressures in aquatic species allow more accessible M2‐like anti‐inflammatory programmes, meanwhile oxygen‐rich environments in terrestrials favour M1‐like inflammatory programmes.^78^, ^79^ Indeed, oxygen flux is shown to be higher in tail regeneration‐incompetent tadpoles.^66^ Overall, the immune system seems to be one of the key contributors to regeneration, and strategies to overcome persistent inflammatory programmes might be required for inducing mammalian limb regeneration.

### 1.3 Inability to generate or mobilise cells and form structures required for regeneration

Another prominent question related to why mammals fail to regenerate their limbs is whether they have the mechanisms to recruit or generate the necessary cells, including stem and progenitor cells, required to regrow the missing structure.

Recent findings emphasise that appendage regeneration primarily uses lineage‐restricted stem and progenitor cells to form a blastema in vertebrates.^2^ Some of the appendage regeneration scenarios use resident adult stem cell populations. One example of this would be the mobilisation of *Pax7*+ resident stem cell populations in axolotl limbs that contribute to the blastema and ultimately regrow muscle.^80^ Alternatively, in some instances, cells can enhance their plasticity to contribute to newly produced cell types. For example, specific fibroblasts trigger a new transcriptional programme enabling them to exhibit multipotency and differentiate into chondrogenic and fibroblastic cells during limb regeneration. Such a response was considered to be a dedifferentiation event as fibroblasts exhibit the features of multipotent limb bud progenitor cells. However, the definition of dedifferentiation is still being heavily discussed (especially when taking into consideration the recent findings from scRNA‐Seq experiments^81^, ^82^, ^83^, ^84^) and it is unclear if this indicates a complete reversion to a progenitor cell state, or simply exhibiting some progenitor‐like characteristics. Thus, in this manuscript, we will mainly discuss enhancing cellular plasticity as a feature for regeneration. From this point, mammals and amphibians share many cellular programmes and cell types such as muscle stem cells, distinct fibroblast populations, mesodermal limb bud progenitors and heterogeneous epithelial layers. In addition, a long list of phenotypes summarised in this manuscript hints that regeneration‐incompetency could be due to the inability of epidermal or mesodermal cells to activate regeneration‐promoting programmes upon amputation.

#### 1.3.1 Inability to form an epithelial signalling center (Epithelial incompetency)

Extensive studies in amphibians and amniotes suggest that one of the main reasons for limb regeneration incompetency is that amniotes cannot induce a mature specialised wound epidermis, AEC.^85^ This notion was based on several historical observations. In classical experiments using salamanders, removing the AEC or blocking AEC formation resulted in limb regeneration‐incompetency.^86^, ^87^, ^88^, ^89^ Meanwhile, amputations and physical damage were found to induce connective tissue mesodermal cells to revert to a phenotype morphologically resembling limb bud progenitor cells, even without an AEC.^4^ These cells alone, however, were not sufficient to regenerate the limb. Second, grafting limb bud mesoderm onto different body regions in *Xenopus* did not result in limb growth. This was only observed when an epidermal covering was present indicating that the interaction between the epithelial tissue and mesenchymal cells are important for limb growth.^90^ Lastly, as *Xenopus* mature and progressively lose the ability to regenerate limbs, AEC formation becomes problematic.^81^, ^91^ Whilst regeneration‐competent tadpoles can form an AEC, this ability decreases in regeneration‐restricted tadpoles and diminishes when tadpoles become regeneration‐incompetent.

In addition to amphibian phenotypes, there were several lines of evidence in amniotes highlighting the importance of epidermal cells in enabling growth, an important aspect of regeneration. Particularly, the AEC, which forms during limb regeneration, has been suggested to function in a similar manner to the apical‐ectodermal‐ridge (AER), a key epithelial structure that directs growth and patterning of the developing limb. Removal of the AER or replacing the AER with back skin during limb development does not result in AER re‐formation and limb development is halted.^92^, ^93^ Thus, having limb bud progenitor cells alone is not sufficient to induce AER formation and limb development. Secondly, removing the distal mesenchyme underlying the AER which contains proliferating limb bud progenitor cells, without removing the AER, results in normal limb development.^94^ Thirdly, dissociated and grafted AER cells were shown to induce some form of regeneration after chicken limb bud amputations.^10^ Similarly, amputating chicken limb buds and combining the stump with the AER, or implanting beads containing AER associated FGF2 results in the formation of distal elements, hence inducing a partial regenerative response.^36^

Despite these findings, direct evidence demonstrating that AEC grafts can restore limb regeneration remains untested in mammals. Moreover, although limb regeneration is linked with an AEC, there are some exceptions to this rule. Some studies have reported that pre‐metamorphic tadpoles which cannot regenerate their limbs are unable to form an AEC, whereas froglets, which respond to injury by forming a spike, can (as assessed by Fgf8+ cells at the amputation plane).^95^, ^96^, ^97^, ^98^, ^99^ This finding may indicate the presence of additional barriers contributing to regeneration‐incompetency in froglets, or froglet and tadpole AEC may not be similar. Further work will be required to assess the impact of the AEC on inducing or maintaining cells to elicit multipotency and their contribution to regeneration.

#### 1.3.2 Inability of mesenchymal tissues to contribute to limb regeneration (Mesenchymal incompetency)

A second hypothesis that explains regeneration incompetency in amniotes is that the fibroblastic mesoderm has lost the ability to initiate a regenerative response.

During limb development, the growth of the limb relies on initial signalling from the mesoderm to induce the formation of an epidermal signalling center, the AER.^93^, ^100^, ^101^ Similarly, during regeneration, should the mesoderm fail to signal appropriately, an AEC will fail to form and regeneration will be impeded. On this basis, *Xenopus laevis* tadpole limb regeneration incompetency may arise as mesodermal cells become intrinsically incompetent to activate regenerative programmes as they mature and become committed to a particular fate during development.^22^, ^23^, ^102^ Here, intrinsic properties may indicate the transcriptional or epigenetic state of cells. Early experiments demonstrated that grafting whole limb buds from tadpoles to post‐metamorphic froglets and amputating them resulted in limb regeneration.^23^ Meanwhile, grafting froglet blastema tissue to tadpole stumps and amputating them did not result in limb regeneration. Notably, as froglet limbs can still regrow a spike, the intrinsic inability was associated with failure to dedifferentiate and exhibit patterning cues. Still, later work in froglets showed that supplementing the patterning cue, *Shh*, was still insufficient to induce full limb regeneration.^103^ Thus, the inability to regenerate full limbs in froglets may not be simply the failure to activate patterning cues.

Further work using recombinant limbs also demonstrated that mesodermal tissue is in part, responsible for regeneration‐incompetency.^102^ In these experiments, researchers combined regeneration‐competent mesoderm and regeneration‐incompetent epidermis. When such recombinant limbs were amputated, they were still able to regrow their lost limbs. Meanwhile, when recombinant limbs are generated from regeneration‐incompetent mesoderm and regeneration‐competent epidermis, tadpoles fail to regrow their limbs. Nonetheless, these studies were largely conducted with tissue level observations involving tissue grafting approaches. Hence, these experiments were unable to discriminate which specific cell causes regeneration‐incompetency or how the local tissue microenvironment contributed to these results.

More recently, the ability of some fibroblasts to induce a multipotency programme as seen in limb bud mesodermal progenitors was suggested to influence regenerative‐outcomes.^104^ Here, limb bud progenitor multipotency is considered to be the ability to differentiate into chondrogenic and fibroblastic lineages. A scRNA‐Seq and grafting based study suggested that froglet fibroblasts fail to activate limb bud genes upon amputation. To test if amputated froglet limb cells can acquire features of tadpole limb bud cells, cells from the froglet blastema were grafted to limb buds of developing tadpoles and found not to exhibit multipotency. Based on this, froglet blastema cells were suggested to fail to revert to a progenitor‐like state and are intrinsically incompetent to activate this programme. However, interestingly, grafting blastema cells from froglets to the froglet blastema environment results in multipotency, arguing that the cells have the multipotency programme but the environment of the developing tadpole limb does not allow cells to exhibit this ability. Indeed, the limb bud develops prior to metamorphosis; meanwhile cells taken from froglet limbs in the post‐metamorphic period are subject to a different immune system, hormonal, and metabolic regulation.^105^, ^106^, ^107^, ^108^ Thus, it remains unclear if the interaction between the post‐metamorphic grafted cells and the pre‐metamorphic limb bud environment resulted in these experimental outcomes or if it was simply due to the grafted cells themselves. For this, the impact of the metamorphosis associated changes on multipotency needs to be investigated.

In parallel to the grafting‐based assays, several studies showing epigenetic alterations in *Xenopus laevis* cells during maturation also support the idea that intrinsic properties limit regeneration capacity. First, analyses of limb buds during homeostasis and following amputation has been suggested to exhibit similar activating and repressive histone marks.^41^ Moreover, alterations to chromatin modifiers could impair regeneration‐competency, proposing epigenetic modulations are important for regeneration. More specifically, enhancer sequences associated with *Shh*, an important regulatory gene required for limb bud development, accumulate more repressive DNA methylation modifications as the *Xenopus laevis* transitions to regeneration‐incompetency.^109^ Meanwhile, the orthologous regions in axolotls, which are able to regenerate throughout their lifetime, remain less methylated. These exciting findings suggest that cells may be changing their epigenetic programmes and not be primed to participate in regeneration. As these studies are conducted with bulk‐tissue analysis, it is still not clear which specific cell types within these tissues show these methylation profiles or if these observations are due to increased heterogeneity within the limb. To answer these questions will require single‐cell assessment of epigenetic features of the limbs.

Contrary to these findings arguing that mesodermal incompetency results in regenerative failure, certain experiments indicate that mesodermal deficiencies by themselves are not sufficient to explain these outcomes. First, neonatal mouse limb amputations result in regenerative failure and grafting embryonic limb bud mesodermal cells to amputated neonatal limbs cannot induce limb regeneration.^9^ Instead, limb bud mesoderm grafts result in disorganised chondrogenic and osteogenic cell growth. Secondly, in salamanders, removing the AEC and grafting back skin to the amputation plane does not result in limb regeneration, although the mesodermal cells would be intrinsically competent for regeneration.^89^ Likewise, removal of the AER during limb development does not enable limb growth, despite the fact that limb bud progenitors would still be present.^92^, ^93^

Cumulatively, these results suggest that regeneration‐incompetency occurs, in part, due to mesodermal cells failing to provide necessary signals to initiate a regenerative response. However, it is largely unclear which intrinsic and extrinsic factors they require to contribute to successful limb growth.

#### 1.3.3 Effects of the local tissue microenvironment on regeneration incompetency

Although the properties of the immune system, epithelium, or mesoderm is long debated, it is unlikely that there is a single causative factor contributing to regeneration‐incompetency. Notably, current observations imply deciphering dynamic cell–cell interactions and the local tissue microenvironment at the amputation plane will be essential to understanding why regeneration fails.

*Xenopus laevis* tadpole limb regeneration is amputation position‐dependent (e.g. amputating through bones results in a less robust regenerative response compared to amputation through joints).^22^, ^34^, ^37^, ^38^ Hence, even if cells undergo intrinsic changes during development, positional dependence highlights that this is likely regulated locally. Recently, mature chondrogenic cells (which are associated with proximal regions of the limbs, and bones) were suggested to secrete inhibitors (e.g. NOGGIN), inhibiting the formation of an AEC and compromising limb‐regenerative ability.^81^ Moreover, treating regeneration‐competent limbs with regeneration‐incompetent secreted factors can block AEC formation, arguing the environmental cues resulting from cell–cell interactions can override intrinsic properties.

Notably, Lin et al. devised one of the most striking strategies to induce limb regeneration.^96^ Grafting engineered *Xenopus* limb bud mesodermal cells with a cocktail of growth factors (e.g. SHH, FGF10), froglets were able to induce growth and patterning reminiscence of full limb regeneration. Some of these froglets were able to grow back limb‐like structures with extremities similar to digits. Meanwhile, grafting limb bud cells alone or individual growth factors failed to induce limb regeneration in adult frogs. Critically, grafted cells were fluorescently labelled in one colour while the host animal cells expressed a different fluorophore. In these intricate experiments, authors noted that in some frogs, it was not just the grafted material, but also the host tissues including mesodermal, epidermal, and muscle lineages that significantly contributed to the regenerated structures. These findings highlight that manipulating the local environment can prompt cells to participate in regeneration and overcome limitations with potential intrinsic features.

In accordance with the findings in *Xenopus*, studies in mice have also highlighted the importance of local microenvironmental cues in regulating regenerative outcomes. Of note, transplanting cells from regeneration‐incompetent regions of the body (e.g. fibroblasts from distal digits or back skin) into regenerating digit tips enabled these cells to acquire a progenitor‐like transcriptional state. Remarkably, these cells then went on to contribute to the newly regenerated tissues of the digit.^84^, ^110^ Moreover, limb bud progenitors or mouse iPSCs derived limb‐bud progenitor‐like cells were grafted to restore proximal digit tip amputations.^111^ In these experiments, both the host and the donor tissues contributed to bone and soft connective tissue growth. These results indicate, that like *Xenopus*, manipulating environmental cues within the tissue may unlock the potential of mammalian appendages to regenerate. In support of this concept, one study has already demonstrated that application of BMP2 or 9 to non‐regenerating distal digit tip amputation wounds can induce bone outgrowth or joint‐like structures.^112^ Collectively, these studies provide evidence that we may ultimately be able to prompt cells to participate in regeneration and overcome potential intrinsic limitations.

#### 1.3.4 Genetic perturbations overcoming Xenopus limb regeneration‐incompetency or inhibiting murine digit tip regenerative capacity

Certain genetic perturbations have been shown to improve *Xenopus* limb regenerative capacity while others inhibit regeneration competency in murine distal digit tips. Here, we will discuss some of these perturbations that may have an association with the above discussed hypotheses.

#### 1.3.5 Xenopus

Efforts to induce limb regeneration have mainly focused on identifying regeneration‐promoting signals that are thought to be less expressed in regeneration‐incompetent stages. As FGF10 was shown to be essential for amniotic AER formation, it was also highly probable that it would play a role in tissue regeneration. Therefore the expression pattern and the role of FGF10 was investigated in *Xenopus*.^102^ Mesodermal FGF10 expression was shown to decrease in accordance with the progressive loss of *Xenopus* limb regeneration capacity,^102^ and supplementing limbs with FGF10 was sufficient to induce AEC formation and restore this ability.^113^ In subsequent studies, FGF10 was found to suppress chondrogenic progression and therefore suggested to operate upstream of the regulatory pathways promoting chondrogenesis (e.g. NOGGIN).^81^ This suggests that FGF10 may not only provide regeneration‐activating signals but also limit regeneration‐inhibitory cues.

Several other signalling cues have been shown to regulate *Xenopus* limb regeneration. Recent studies have demonstrated that B‐catenin overexpression was shown to improve *Xenopus* limb regeneration by aiding in the formation of the AEC.^114^ Meanwhile, overexpression of the Wnt inhibitor *Dkk1*, inhibits AEC formation without affecting FGF10 and other gene expression in the distal mesenchyme.^115^ Furthermore, unlike the tadpole limb regeneration phenotype, *Dkk1* overexpression does not impair spike formation in froglets.^116^ Meanwhile, overexpression of *Noggin* blocks both tadpole limb regeneration and froglet spike formation.^3^, ^117^ Lastly, overexpressing *Msx1*, which is mostly associated with a dedifferentiation phenotype, fails to induce *Xenopus* tadpole limb regeneration, although it can create a pulse of blastemal and epithelial growth.^118^ Although *Msx1* overexpression fails to induce limb regeneration at ‐incompetent stages, it does improve amputation‐outcomes in regeneration‐restricted tadpoles. Interestingly, *Msx1* and *Noggin* co‐overexpression abolish this positive effect, and resulting in regeneration failure.^118^ These findings provide additional evidence arguing that activating signals and lack of inhibitory cues are critical for regeneration, and suggest that environmental modulation can be used to induce limb regeneration.

In addition to the above phenotypes, several other pathways were shown to induce limb regeneration including manipulation of Dicer,^119^ the NF‐kB pathway,^68^ and the melanocortin 4 receptor (Mc4r), pathway.^120^ However, it remains unclear if these perturbations might be resulting in dysregulation of the immune system, altering intrinsic abilities or changing environmental cues that favour regeneration. In the future it will be essential to address how these molecular players intersect to develop an integrated model for regeneration‐competency and ‐incompetency.

#### 1.3.6 Mouse

While the mammalian distal digit tip appears to regenerate in the absence of an observable AEC, there are several studies highlighting that epithelial tissues play a critical role in facilitating the regenerative process. First, a seminal study by Takeo and colleagues (2013) explored the function of nail epithelial cells during murine digit tip regeneration, since the digit will only regenerate if part of the nail remains.^15^ Here, it was demonstrated that canonical Wnt signalling in the nail epidermis is important for instructing the underlying mesenchymal response during regeneration. Upon amputation of distal digits, mice with conditional deletion of beta catenin from the nail epithelium failed to reinnervate, form a blastema and regenerate bone.^15^ Interestingly, however, inducing Wnt expression in epithelial tissue of the skin from proximal amputations was not sufficient to overcome regeneration incompetency.^15^ This indicates that the nail likely supports tissue regeneration through multiple mechanisms that are as yet unknown. Second, disrupting the wound epidermis in neonatal mice, inhibited distal digit tip regeneration.^7^ While the mechanisms behind this regenerative failure were not investigated, a subsequent study has demonstrated that the wound epidermis expresses the chemoattractant, cytokine stromal derived factor 1a (SDF1‐a).^121^ Mice treated with ADM3100, a chemical that blocks the receptor for SDF1‐a presented with deficits in blastema cell migration and partial inhibition of bone growth. Collectively, these perturbations demonstrate that the epithelium within the digit tip plays an important role in coordinating the regenerative response.

There are several other signalling pathways, predominately expressed within the mesenchymal cells of the digit, that have been shown to impact mammalian digit tip regeneration. Of note, BMP signalling has been shown to enhance cell recruitment to the blastema and facilitate bone growth.^121^, ^122^, ^123^ Conversely, treating amputated distal digit tips with the BMP antagonist Noggin, inhibits regeneration.^123^ Meanwhile treatment of proximal amputations with BMP2 or BMP7 or sequentially with BMP2 followed by BMP9, induced longitudinal regrowth of the middle phalanx and partial regeneration of the joint.^112^, ^123^, ^124^ In addition to BMP signalling, embryonic mice carrying targeted deletions of *Msx1* were found to display deficits in digit tip regeneration.^122^ The importance of *Msx1* to digit tip regeneration may, however, be age‐dependent. This is because *Msx1* is not expressed in the blastema of neonatal mice^7^, ^122^ and is indiscriminately expressed in mesenchymal tissues of both regenerative and non‐regenerative amputations in adult mice.^84^ The function of *Msx1* during digit tip regeneration in older mice has yet to be determined. Lastly, mice deficient in Mc4r, which plays a pivotal role in regulating energy balance, fail to regenerate their digits upon amputation.^120^ This may be due, in part, to an inability to attract nerves that promote blastema growth and subsequent digit tip regeneration.^125^ Altogether, these perturbation studies support the concept that instructive epithelial tissues and responsive mesenchymal cells are essential to ensuring regeneration competency.

## 2 CONCLUDING REMARKS: EVOLUTIONARY LOSS OF REGENERATIVE CAPACITY

Current evidence strongly argues that mammals contain the necessary cells or transcriptional programmes to mediate regeneration. On the contrary, the regulation of these cells and the multi‐phasic processes that govern during regeneration may be different between species, suggesting that there could be multiple barriers inhibiting mammalian limb regeneration.

Cross‐species comparison between regeneration‐competent and ‐incompetent species can pave the way to identifying and eliminating these potential barriers. From this point, recent research underscores that mammalian and salamander limb development are significantly different. Meanwhile, *Xenopus laevis* limb development is more similar to mammals and can serve as a bridge between the highly‐regenerative salamanders and regeneration‐incompetent amniotes.^126^ The recent surge in single‐cell ‐omics approaches provide a novel opportunity for cross‐species comparisons. However, animals exhibit many differences including differences in their humoral immune system response, living conditions, and even the cellular composition of limbs show variability. Indeed, the identified regeneration‐competent species are aquatic, while incompetent species are terrestrial. Such environmental changes may exert their impact on oxygen influx, mechanical properties, microbiome, metabolic requirements, and more. Altogether, there are significant differences between species that could account for differences in regenerative capacities, and the current ‐omics methods can only target limited aspects (e.g. transcriptomics, chromatin accessibility) of these. Based on current results, it is highly likely that regeneration‐incompetency stems from multiple changes within different cellular lineages. Studying how cell–cell interactions are resolved in time and space will be the next critical step to unlocking the potential of mammalian limbs to regenerate.

Although it remains unclear how regenerative abilities are selected or preserved during evolution, the findings discussed here speculate how mammals may have lost their regeneration competency. For a long time, limb regenerative ability was thought to be unfavourable for survival in nature, as limb regeneration requires an open wound prone to infections rather than a rapid fibrotic scarring response. Moreover, slowly regrowing limbs may create an imbalance in mobility and a high demand on energy stores. Hence, inflammatory scarring programmes could be advantageous for avoiding predators. Although the lower vertebrates tend to exhibit higher regenerative abilities, it is not clear why cells in higher vertebrates would be shifting their intrinsic properties away from a regenerative response, and how such a shift may be more advantageous for survival in nature. Recently, chondrogenesis‐associated secreted factors are suggested to be a contributor to regeneration‐incompetency through its impact on blocking AER cell formation in *Xenopus*.^81^ This suggestion conveys new perspectives on limb regeneration incompetency. Particularly, the pace of limb chondrogenesis shows a drastic difference between regeneration‐competent aquatic animals and terrestrial regeneration‐incompetent amniotes. Specifically, limb chondrogenesis occurs in only 3–4 days in amniotes; meanwhile, it takes at least a month in amphibians.^127^, ^128^ Indeed, a detailed analysis on the progression of chondrogenesis and ossification in the axolotl has been shown to be completed over the course of a year.^128^ Further evidence to support this can be seen when correlating pace of chondrogenesis with limb regenerative ability. For example, bone fractures heal faster in mammals than aquatic regeneration‐competent amphibians,^129^, ^130^ although further studies with standardised approaches are needed. One potential speculation could be that terrestrials may require a more robustly working skeletal system to be advantageous on land. Meanwhile, aquatic animals may not prioritise such an ability, as swimming behaviour would not demand a similar reliance on a functioning skeletal system.

Current studies argue that the immune system and mesenchymal and epidermal cells could separately contribute to regeneration‐incompetency. Moreover, genetic perturbations restoring regenerative abilities might be operating in parallel. Nonetheless, further work integrating how different cell types and various molecular pathways interact will be critical to reveal how regeneration could be restored in higher vertebrates, including humans.

## CONFLICT OF INTEREST

The authors declare no competing financial interests.

## FUNDING INFORMATION

C.A. is supported by the EPFL School of Life Sciences, and the Fondation Gabriella Giorgi‐Cavaglieri, and M.A.S. is supported by the Wellcome Trust/MRC Cambridge Stem Cell Institute starter grant funding.

## AUTHOR CONTRIBUTIONS

C.A., and M.A.S. wrote the manuscript. M.A.S and C.A. curated the Figures. M.A.S. drew the most of the figures.

## Data Availability

Data sharing not applicable to this article as no datasets were generated or analysed during the current study.
